# Autoimmune Diseases and the Anterior Segment of the Eye

**DOI:** 10.1155/2018/6029460

**Published:** 2018-11-01

**Authors:** Marta Sacchetti, Flavio Mantelli, Alessandro Lambiase

**Affiliations:** ^1^Department of Sense Organs, Sapienza University, Viale del Policlinico 155, 00196 Rome, Italy; ^2^Department of Biology, College of Science and Technology, Temple University, 1900 N 12th Street, Philadelphia, PA 19122, USA

This special issue focuses on the current approaches for diagnosis, evaluation, and management of autoimmune diseases of the anterior segment of the eye, which range from immune keratoconjunctivitis to anterior uveitis. Immune diseases of the anterior segment of the eye may be caused by several local or systemic conditions and may present in a wide range of diseases including dry eye syndrome, ocular cicatricial pemphigoid (OCP), graft versus host disease (GVHD), and some forms of anterior uveitis often associated with systemic autoimmune diseases such as ankylosing spondylitis. These conditions represent some of the most difficult conditions to diagnose and manage in ophthalmology and often require a multidisciplinary approach.

Specifically, in this special issue, Tao and colleagues evaluated reliability and validity of the most commonly used quality of life questionnaires in Chinese patients with dry eye disease; Szepessy et al. evaluated the alterations of central retinal and choroidal thickness and the severity of anterior chamber inflammation in patients with acute anterior uveitis associated with seronegative spondyloarthropathy; Qui et al. described the characteristics of ocular manifestations of a large cohort of patients with a diagnosis of acute or chronic ocular GVHD; Nebbioso et al. evaluated the potential role of vascular endothelial growth factor (VEGF) and mucins in patients with vernal keratoconjunctivitis (VKC). In addition, Leuci et al. reported the long-term clinical outcome of 6 patients with OCP treated with intravenous immunoglobulin therapy (IVIg), which maintained remission of the disease and did not show progression, for a total follow-up period of 9 years after the end of IVIg treatment.

The topics presented in this special issue evaluated few aspects of the large and heterogeneous group of disorders which may be included in the group of autoimmune diseases of the anterior segment of the eye. However, it is clear that these conditions require a multidisciplinary approach and often represent a challenge for clinicians due to the lack of specific diagnostic criteria and specific treatments. In fact, most immune diseases are currently treated with local or systemic corticosteroids or immunosuppressive drugs, which may be effective in controlling the inflammatory reaction but are commonly associated with important local and systemic side effects. By this point of view, the increasing understanding of novel pathogenic mechanisms of autoimmune diseases will lead to the development of novel, more specific drugs designed to target the molecules involved in the inflammatory reaction.

In addition, autoimmune diseases of the anterior segment of the eye may have complications that extend beyond the anterior segment and can impair visual function. In fact, visual function may be directly impaired in dry eye disease or immune keratoconjuctivits due to corneal damage and scarring, or, alternatively, other autoimmune diseases such as anterior uveitis may induce glaucoma with damage to the optic nerve or macular changes, which cause decrease of visual acuity. Not only the diseases but also some of their treatments may induce long-term adverse effects impairing visual function; for example, chronic use of corticosteroid eye drops in patients with immune diseases of the eye is a well-known cause of cataract and glaucoma.

Based on these considerations, it is clear that a further progress in understanding the pathogenesis of ocular immune reactions is highly sought after, together with more standardized diagnostic procedures and protocols for several autoimmune diseases of the anterior segment of the eye. Such progresses will hopefully also lead to the development of novel and more targeted therapeutic approaches that can improve clinical outcome and quality of life of patients with these diseases.

